# Combining spectral and wavelet texture features for unmanned aerial vehicles remote estimation of rice leaf area index

**DOI:** 10.3389/fpls.2022.957870

**Published:** 2022-08-04

**Authors:** Cong Zhou, Yan Gong, Shenghui Fang, Kaili Yang, Yi Peng, Xianting Wu, Renshan Zhu

**Affiliations:** ^1^School of Remote Sensing and Information Engineering, Wuhan University, Wuhan, China; ^2^Lab for Remote Sensing of Crop Phenotyping, Wuhan University, Wuhan, China; ^3^College of Life Sciences, Wuhan University, Wuhan, China

**Keywords:** leaf area index (LAI), vegetation index (VI), unmanned aerial vehicle (UAV), remote sensing (RS), texture, wavelet, rice

## Abstract

Estimating the crop leaf area index (LAI) accurately is very critical in agricultural remote sensing, especially in monitoring crop growth and yield prediction. The development of unmanned aerial vehicles (UAVs) has been significant in recent years and has been extensively applied in agricultural remote sensing (RS). The vegetation index (VI), which reflects spectral information, is a commonly used RS method for estimating LAI. Texture features can reflect the differences in the canopy structure of rice at different growth stages. In this research, a method was developed to improve the accuracy of rice LAI estimation during the whole growing season by combining texture information based on wavelet transform and spectral information derived from the VI. During the whole growth period, we obtained UAV images of two study areas using a 12-band Mini-MCA system and performed corresponding ground measurements. Several VI values were calculated, and the texture analysis was carried out. New indices were constructed by mathematically combining the wavelet texture and spectral information. Compared with the corresponding VIs, the new indices reduced the saturation effect and were less sensitive to the emergence of panicles. The determination coefficient (R^2^) increased for most VIs used in this study throughout the whole growth period. The results indicated that the estimation accuracy of LAI by combining spectral information and texture information was higher than that of VIs. The method proposed in this study used the spectral and wavelet texture features extracted from UAV images to establish a model of the whole growth period of rice, which was easy to operate and had great potential for large-scale auxiliary rice breeding and field management research.

## Introduction

Rice is the staple food for more than 50% of the global population (Yuan, 2014). A useful index to monitor crop growth is the leaf area index (LAI), which is defined as the total leaf area per unit ground area. LAI is commonly used as an important parameter for photosynthesis, respiration, and productivity of vegetation (Gower et al., 1999; Liang et al., 2015). In the practical application of precision agriculture, LAI is also an effective indicator for diagnosing crop growth, estimating biomass, and predicting yield (Liu et al., 2005; Zhou et al., 2017; Fang et al., 2019). Therefore, it is very important to obtain crop LAI accurately, which can obviously improve the monitoring of crop growth and yield prediction in agricultural remote sensing.

Although the crop measurements based on the traditional manual methods are effective, they are labor-intensive and time-consuming. Therefore, it is not possible to quickly obtain temporal and spatial information on crops in detail on a large scale. The remote sensing technique, which is non-destructive and often applied at large scale, has achieved satisfactory results in the estimation of crop LAI and becomes more and more important. There are two main techniques to estimate LAI by remote sensing images: empirical statistical models (ESMs) (Zarate-Valdez et al., 2012) and radiative transfer models (RTMs) (Dorigo, 2012). Although RTM approaches can simulate the radiation transmission process of the optical signal in the canopy, they need many input parameters and high computational costs. In general, regression models are used to establish the relationship between LAI and vegetation indices (VIs). Numerous optical VIs are calculated from the combinations of two or more different spectral bands, mostly distributed in the visible and near-infrared regions of the spectrum. Through band combinations, the VI can highlight vegetation information which is in the reflectance spectrum, inhibit the effects of other interfering factors such as the structure of leaves and canopy (Herrmann et al., 2011; Verrelst et al., 2015), and reduce the soil, atmosphere, and solar–target–sensor geometry effects as much as possible (Moulin, 1999; Matsushita et al., 2007; Viña et al., 2011). In the 1970s, the normalized difference vegetation index (NDVI) and ratio vegetation index (RVI) were first applied by Rouse for estimating vegetation growth characteristics (Rouse et al., 1974). In recent years, many VIs have been applied in the estimation of LAI (Fang et al., 2019). Viña et al. (2011) evaluated several VIs to estimate the green leaf area index of corn and soybean, in which the chlorophyll index (CI_*green*_, MTCI, and CI_*red  edge*_) showed a very significant linear relationship with green LAI. Qiao et al. (2020) used six VIs derived from MERIS data to establish LAI seasonal tracks for different types of vegetation, including deciduous forest, evergreen forest, and crops. Dong et al. (2019) proved the potential of VIs based on red-edge reflectance derived from multi-temporal RapidEye images in the LAI estimation of spring wheat and rape. Indices that incorporated the reflectance of red-edge bands had increased potential for estimating LAI. Liang et al. (2015) proposed a hybrid inversion method for estimating the crop LAI and evaluated 43 VIs to determine the best VIs in the estimation of LAI. However, there are still some problems to be settled in the estimation of rice LAI.

The changes in the canopy morphology and structure of rice are more obvious and complex than those of other vegetation types in different growth periods (Sakamoto et al., 2011; He et al., 2019). Rice seeds are transplanted into soil or water after germination. With the tillering and jointing of rice, the leaves gradually increase in size and leaf area. At this time, the canopy is blocked by leaves, and the soil background is almost invisible. When 20% of panicles have exserted from the sword leaf sheath, the plants enter the heading stage. The rice panicles are slender and rough in shape. When the rice enters the ripening stage, the panicles begin to droop and change color from green to golden. Meanwhile, the leaf begins to wither and falls from bottom to top, which makes the canopy structure of the crop more complex (Reza et al., 2019; Zha et al., 2020). The effect of remote estimation of rice LAI during the whole growth stage is greatly affected by the sophisticated changes in rice phenology.

In the early growth stage, the canopy reflectance is strongly influenced by the soil background (Zarco-Tejada et al., 2001; Croft et al., 2014; Tian et al., 2014). When the rice canopy closes, VIs tend to saturate in high coverage conditions (Viña et al., 2011). After the heading stage, the panicles begin to appear and are disorderly distributed. Remote sensing reflectance data contain not only leaf information but also panicle information. At the same time, the leaves begin to wither and yellow, and the canopy spectrum is greatly affected (Yang Q. et al., 2022). The VIs are very easy to be saturated in the late growth stage of rice, and LAI is relatively stable from the tillering to the jointing stage, but its value range is wide. Therefore, it is necessary to estimate LAI accurately in the later stages of growth. Wang et al. (2019) estimated LAI of rice before and after heading stages using 10 VIs. It was found that VARI was susceptible to the emergence of panicles, and the accuracy of estimation of LAI was affected by the panicles. Zhou et al. (2017) also found that panicles reduced the accuracy of grain yield estimation at the late growth stages of rice. Therefore, the model of rice LAI estimation without considering the variability of different fertility periods is flawed and ignores the complexity of rice. Differences in the canopy structure during rice growth must be taken into account to make more accurate estimates. In addition, for professionals, observing whether paddy plots are heading is a laborious and time-consuming job. Consequently, the estimation of rice LAI for the whole growing season is important.

Texture analysis plays an important role in image processing and is usually used to define the variability of pixel values between adjacent pixels of the analysis window (Kelsey and Neff, 2014). In recent years, many studies have considered image texture features when estimating LAI (Zhang et al., 2021, 2022), AGB (Hlatshwayo et al., 2019; Yue et al., 2019), N nutrition parameters (Zheng et al., 2020), and plant potassium accumulation (PKA) (Lu et al., 2021). Li S. et al. (2019) used the UAV and RGB images to build the NDTI model. The indices based on the RGB images were combined with the calculated the NDTI to improve the accuracy of rice LAI estimation. Duan et al. (2019) proposed a method using Fourier spectral energy percentage (FSEP) and found that the FSEP extracted from VI images could obtain a more accurate rice LAI estimation model than the VI. In addition, the VI is highly susceptible to saturation in late rice growth stages, resulting in unsatisfactory LAI estimation. The combination of texture features and VIs is helpful in improving the accuracy of LAI estimation. However, the existing research methods cannot accurately reflect the complex changes in the rice canopy structure at different growth stages. On this foundation, it is necessary to find a texture feature that can adapt to multi-temporal changes of rice to assist the VI to improve the sensitivity of LAI estimation, especially in the canopy closure stage and post-heading stage. Wavelet texture features can quantify the differences in image texture features (Ren et al., 2021) and reveal the differences in the canopy structure in the process of rice growth.

In recent years, wavelet analysis with time–frequency and multi-resolution characteristics has become an effective tool for texture analysis (Huan and Hou, 2008), and it has been widely used for feature extraction (Ghazali et al., 2007), image denoising (Gökdağ et al., 2019), and object/tissue detection (Jain and Salau, 2019). The image is decomposed by the two-dimensional (2D) discrete wavelet transform (DWT) into four sub-bands: LL, reflecting the approximate information of the image, and LH, HH, and HL, reflecting vertical, diagonal, and horizontal detail information of the image, respectively. Most of the energy is concentrated in the LL sub-band, which mainly reflects the approximate component information of the original image (Chang and Kuo, 1993; Bruce et al., 2002; Quandt et al., 2015). The DWT, which is considered to be an efficient method for extracting hyperspectral features, is applied to quantify pigment concentration (Blackburn, 2007), retrieve soil moisture (Peng et al., 2013), and estimate LNC (Li F. et al., 2019) and crop residue quality (Sahadevan et al., 2014). In addition, energy is often used as a texture feature extracted by wavelet transform (Salari and Ling, 1995; Banskota et al., 2013), which can represent texture features in space from the point of energy distribution in the frequency domain and has great texture representation capability (Ren et al., 2021). The combination of texture features and spectral features has shown great potential in LAI estimation (Zheng et al., 2020). Wavelet texture features can enhance the discrimination ability of spatial information (Eckert, 2012). VIs contain rich spectral information, and wavelet texture features can provide spatial structure information of multiple growth stages of rice. Therefore, the advantage of wavelet texture features in capturing the difference in the canopy structure at different growth stages makes it a suitable index to improve the accuracy of rice LAI estimation combined with spectrum features.

Unmanned aerial vehicles (UAVs) have developed rapidly in recent years and have become a promising technology in disaster rescue, transportation, agriculture, and environmental monitoring (Alsamhi et al., 2021, 2022; Saif et al., 2021; Amarasingam et al., 2022; Yang Z. et al., 2022). UAV images are also applied in precision agriculture due to high resolution and low cost. For example, Jin et al. (2017) introduced a method to estimate wheat density using UAV images obtained at very low altitudes. Pádua et al. (2018) conducted multi-temporal monitoring of vineyard vegetation using UAVs equipped with consumer-grade RGB sensors. High-resolution UAV images can also help extract texture features, so it is necessary to consider texture features when monitoring vegetation growth.

In this study, a method for LAI estimation by combining spectral features and wavelet texture features is developed, given the differences in the rice canopy structure at different growth stages. The main purpose of this study is to discuss the potential of the combination of wavelet texture features and spectral features on the LAI estimation throughout the entire growth stage of rice based on UAV images, which have low cost, high flexibility, and high resolution.

## Materials and methods

### Study area

**FIGURE 1:**
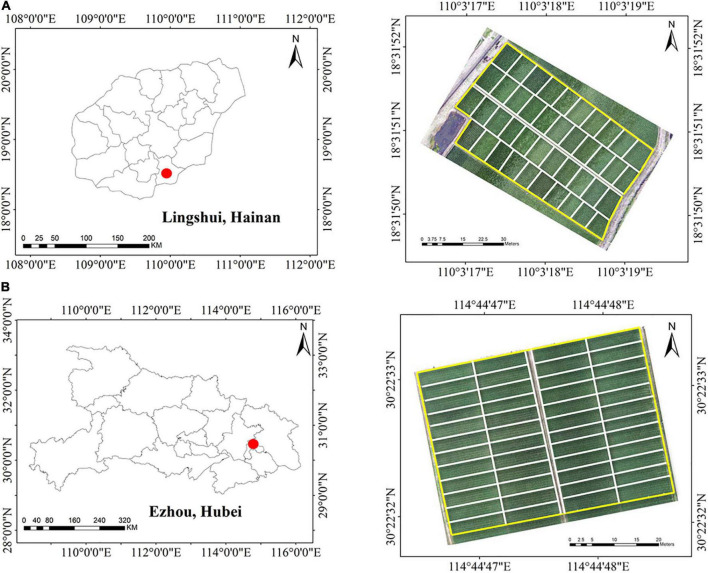
Two experiments were conducted in two study areas. **(A)** Experiment 1 in Hainan. **(B)** Experiment 2 in Hubei.

The two experiments were carried out in two different study areas: One was located in the hybrid rice experiment base of Wuhan University in Lingshui, Hainan, China (18°31′47″N, 110°03′34″E), and the other was located in Ezhou, Hubei, China (30°22′31″N 114°44′50″E) (Figure 1). Except for rice varieties, field management was the same for the two experiments. The planting density was 22.5 bundles per square meter, and the fertilizer supply was 12 kg per hectare. Moreover, in order to ensure the normal growth of the rice plant, the corresponding irrigation measures were carried out in the key growth period. In total, 42 varieties were selected for the experiment in Lingshui, and 48 varieties were selected for the experiment in Ezhou. Each cultivar was placed on the corresponding field plot, and several eye-catching whiteboards were placed at the outer edge of each plot. These whiteboards were used to help us identify different fields and rice varieties in the acquired UAV images. To minimize the effect of destructive sampling on remote sensing canopy spectral features, both experiments divided each hybrid sample plot into a subplot for non-destructively extracting spectral information and a subplot for destructively sampling LAI. The date of LAI sampling, UAV flight, and transplanting of the two experiments are shown in Table 1.

**TABLE 1:** Details of data acquisition for the two experiments.

Experiment	Location	Year	DAT	Growth stage	UAV images	LAI sampling
Experiment 1	Hainan	2018	27,48	Tillering	2,26 February	4,25 February
			60	Jointing	11 March	9 March
			70	Booting and heading	18 March	19 March
			82,99	Ripening	1,15 April	31 March, 17 April
Experiment 2	Ezhou	2019	17,23,27,37,42,47	Tillering	26 June, 2,6,14,22,27July	26 June, 2,6,16,21,26 July
			53,58	Jointing	1,6 August	1,6 August
			63,69,73	Booting and heading	11,16,22August	11,17,21 August
			78,85	Ripening	29 August, 3 September	26 August, 3 September

Experiment 1 lasted for three months in Hainan, with the rice plants transplanted in February 2018 and harvested in April 2018. Hainan has a tropical monsoon climate, with high temperatures the whole year. Lingshui has higher temperatures than the mainlands of China in winter, so it is suitable for rice overwintering growth. We selected 42 rice cultivars for sowing on 10 December 2017. The seedlings were transplanted on 8 January 2018 to 42 plots, each of which covered an area of about 63 m^2^.

Experiment 2 in Hubei lasted for 4 months and was carried out from June to September 2019. Ezhou has a subtropical monsoon climate, and the annual average temperature is 15°C. We selected 48 varieties for sowing on 11 May 2019. The seedlings were transplanted on 9 June 2019 to 48 plots, each of which covered an area of about 36 m^2^.

### Measurements of leaf area index

**FIGURE 2:**
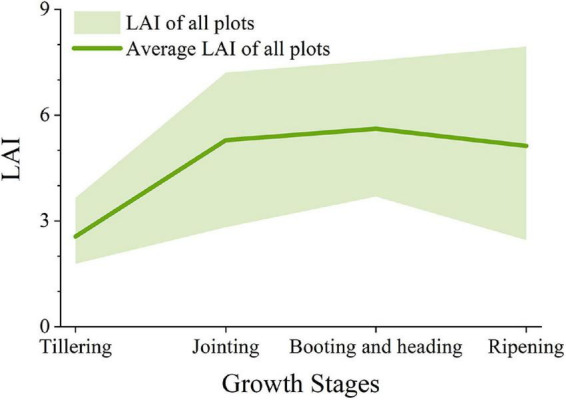
Variation in LAI at different growth stages.

During the entire growth process of rice, we destructively sampled each plot to obtain LAI data of rice. We randomly dug out three bundles of rice plants from the soil in the sampling area, and the sampling process was the same for each plot. The excavated rice plants were quickly put in a bucket and brought back to the laboratory. The samples brought back to the laboratory were measured immediately to obtain LAI data of rice and recorded accurately and in detail. The rice plants were measured after certain treatments to ensure that green leaves were separated from other components, especially withered and yellow leaves and panicles. We used LI-3100C (LI-COR Corporate, Lincoln, NE, United States) to obtain the leaf area of the measured plants. In order to calculate the plot-level LAI, the average area of the three bundles of plants was calculated as the leaf area (LA) of a single plant in each plot: $L\,A\,I=\frac{L\,A}{3}\times \rho$, where LA was the leaf area of all three bundles of rice and ρ was the planting density with 22.5 bundles per square meter. The variation of LAI with the growth period for all fields is shown in Figure 2. LAI of all fields gradually increased in the early growth stage, slowed down at the booting and heading stages, and began to decrease slightly at the ripening stage. LAI changed sharply from the tillering to the jointing stage. However, LAI was relatively stable from the jointing to the ripening stage. The value range was wide, and LAI varied from 2 to 8 at the ripening stage.

### Unmanned aerial vehicle image acquisition and processing

The Mini-MCA 12 multispectral camera (Tetracam Inc., Chatsworth, CA, United States) mounted on an M8 UAV (Beijing TT Aviation Technology Co., Ltd., Beijing, China) was used to obtain multispectral images of the two study areas during the entire growth period of rice. The Mini-MCA had 12 bands with different bandwidths (Table 2), which were mainly in the visible and near-infrared regions and contained abundant information on vegetation.

**TABLE 2:** Bandwidth of the 12 bands of the Mini-MCA camera.

Spectral band	Center wavelength (nm)	Band width(nm)	Spectral band	Center wavelength (nm)	Band width (nm)
Blue	490	10		700	10
	520	10	Red edge	720	10
Green	550	10	NIR	800	10
	570	10		850	10
Red	670	10		900	20
	680	10		950	40

For the corresponding pixels of each lens to overlap on the same focal plane in space, we registered each camera in the laboratory before the flight. The UAV was equipped with a gimbal stable platform to assist the UAV in normal flight and ensure the quality of the images captured. In order to reduce the influence of the changes in the solar altitude angle on the experiments, we carried out every flight from 10 a.m. to 2 p.m. local time. In sunny and windless weather, the UAV was used to collect remote sensing images to avoid the impact of clouds on the images. The flight height of the two experiments was 100 m, and the spatial resolution was 5.5 cm/pixel.

A total of eight calibration targets with stable reflectance values of 0.03, 0.06, 0.12, 0.24, 0.36, 0.48, 0.56, and 0.80, respectively, were observed by the MCA system. Since in this study, the weather was clear during the flight, the DN values of the target and their corresponding reflectance values were used to establish the linear correction equation. The reflectance data of the image were obtained by using the established linear correction equation, thus converting the original images with DN values into reflectance images (Farrand et al., 1994; Fang et al., 2016).

Then, the reflectance images of different bands were used in band mathematics to obtain VI images. The VI was calculated by mathematical combinations between several different bands, which can highlight vegetation features contained in the spectrum. The formula of VIs used in the experiment is given in Table 3, and these indices have been widely used in LAI estimation and biomass prediction. For each plot, a rectangle of appropriate size that reflected the information about the plot was defined. The average of all pixels in the defined rectangle was calculated. Thus, we obtained the plot-level values of the canopy reflectance and VI for subsequent calculations.

**TABLE 3:** VIs used in this study.

VI	Formula	References
Red-edge Chlorophyll Index (CI_*red edge*_)	*R*_800*nm*_/*R*_720*nm*_−1	Gitelson et al., 2014
Green Chlorophyll Index (CI_*green*_)	*R*_800*nm*_/*R*_550*nm*_−1	Gitelson et al., 2014
Normalized Difference Vegetation Index (NDVI)	(*R*_800*nm*_−*R*_670*nm*_)/(*R*_800*nm*_+*R*_670*nm*_)	Rouse et al., 1974
Green Normalized Difference Vegetation Index (GNDVI)	(*R*_800*nm*_−*R*_550*nm*_)/(*R*_800*nm*_+*R*_550*nm*_)	Gitelson et al., 1996
Normalized Difference Red-edge Vegetation Index (NDRE)	(*R*_800*nm*_−*R*_720*nm*_)/(*R*_800*nm*_+*R*_720*nm*_)	Fitzgerald et al., 2010
MERIS Terrestrial Chlorophyll Index (MTCI)	(*R*_800*nm*_−*R*_720*nm*_)/(*R*_720*nm*_−*R*_670*nm*_)	Dash and Curran, 2010
Wide Dynamic Range Vegetation Index (WDRVI)	(α×*R*_800*nm*_−*R*_670*nm*_)/(α×*R*_800*nm*_+*R*_670*nm*_),α=0.2	Gitelson, 2004
Two-band Enhanced Vegetation Index (EVI2)	2.5×(*R*_800*nm*_−*R*_670*nm*_)/(1+*R*_800*nm*_+2.4×*R*_670*nm*_)	Jiang et al., 2008
Visible Atmospherically Resistant Index (VARI)	(*R*_550*nm*_−*R*_670*nm*_)/(*R*_550*nm*_+*R*_670*nm*_)	Gitelson et al., 2002
Optimized Soil-Adjusted Vegetation Index (OSAVI)	(1+0.16)×(*R*_800*nm*_−*R*_720*nm*_)/(*R*_800*nm*_+*R*_720*nm*_+0.16)	Rondeaux et al., 1996
Simple Ratio Index (SR)	*R*_800*nm*_/*R*_550*nm*_	Jordan, 1969; Xue et al., 2004
Ratio Vegetation Index (RVI)	*R*_800*nm*_/*R*_720*nm*_	Pearson and Miller, 1972; Zhou et al., 2017

### Wavelet spectrum texture extraction

In recent years, wavelet analysis has increasingly become an effective technique to analyze textures with its multi-resolution characteristics and its capability to simultaneously represent local features of signal in the time–frequency domain (Huan and Hou, 2008). In this study, the discrete wavelet transform (DWT) was used to process the MCA images and extract the energy texture features, which were combined with VIs to establish a rice LAI estimation model.

The energy of the wavelet function Ψ(*t*) can decay quickly to 0. If Ψ(*t*) is scaled and panned, we can obtain a series of functions (Wang and Du, 2019):


(1) $$\Psi_{a,b}\left(t\right)=|a{|}^{-0.5}\Psi \left(\frac{t-b}{a}\right)\left(a,b\in R,a\neq 0\right)$$

where *a* refers to the scale factor and *b* refers to the shift factor. If *f*(*t*) ∈ *L*^2^(*R*) is the signals with finite energy, the continuous wavelet transform (CWT) function *W*_*f*_(*a*,*b*) is defined as follows:


(2) $$W_{f}\,\left(a,b\right)=\int_{-\infty }^{\infty }f\,\left(t\right)\,\bar{\Psi_{a,b}\,\left(t\right)}\,dt$$

where $\bar{\Psi_{a,b}\,\left(t\right)}$ is the complex conjugate function of Ψ_*a*,*b*_(*t*). The discrete wavelet transform (DWT) discretizes *a* and *b*, $a=a_{0}^{m}\left(a_{0}>1\right)$, $b=nb_{0}a_{0}^{m},\left(b_{0}\in R,\left(m,n\right)\in Z^{2}\right)$. Generally, *a*_0_=2,*b*_0_=1. For signals with finite energy *f*(*t*) ∈ *L*^2^(*R*), the discrete wavelet transform (DWT) function *D*_*f*_(*m*,*n*) is defined as follows:


(3) $$D_{f}\,\left(m,n\right)=\int_{-\infty }^{\infty }f\,\left(t\right)\,\bar{\Psi_{m,n}\,\left(t\right)}\,dt$$

where $\bar{\Psi_{m,n}\,\left(t\right)}$ is the complex conjugate function of Ψ_*m*,*n*_(*t*).

The discrete wavelet transform (DWT) is usually applied to extract relevant information by decomposing the signals into different frequency bands with different resolutions for further analysis. In this study, we used the level 1 Haar wavelet transform to extract texture information by decomposing the rice reflectance images. The Haar wavelet can be defined as follows (Kausar et al., 2019):


(4) $$\Psi \,\left(t\right)=\left\{ \begin{array}{c} 1;0\leq t\leq \frac{1}{2}\\ -1;\frac{1}{2}\leq t<1\\ 0;o\,t\,h\,e\,r\,w\,i\,s\,e \end{array}\right.$$

**FIGURE 3:**
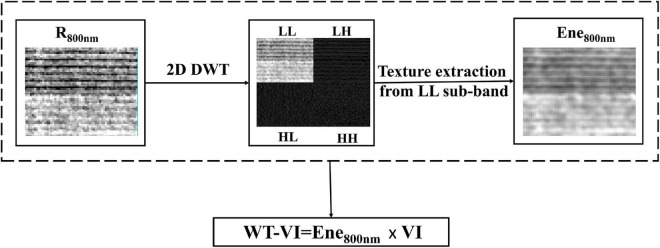
Process of calculating WT-VIs. R_800 nm_ means reflectance of 800 nm. Ene_800 nm_ represents the energy texture features extracted by the wavelet transform.

Compared with other wavelet transforms, the level 1 Haar wavelet is computationally simpler and more efficient in decomposing 2D images, and it has a high speed (Ghazali et al., 2007). The image is decomposed by the 2D discrete wavelet transform (DWT) into four sub-bands: LL, reflecting the approximate information of the image, and LH, HH, and HL, reflecting vertical, diagonal, and horizontal detail information of the image, respectively. Rows and columns are filtered at the same time, where H corresponds to high-pass filtering and L corresponds to low-pass filtering. The rice reflectance images and the sub-band images decomposed by level 1 Haar wavelet transform are shown for reference in Figure 3. Most of the energy of the image was concentrated in the LL sub-band and was very high (Shukla et al., 2022). The LL sub-band reflected the outline of the image and contained most of the information of the image. Therefore, using the texture features extracted from the LL sub-band for LAI estimation can give better results.

The energy feature was extracted from the LL sub-band obtained by wavelet decomposition (Ren et al., 2021). In addition to the textural information, this feature was also able to amplify the dissimilarities that may be present between two regions and represent texture features in space from the point of energy distribution in the frequency domain with less computation and strong texture representation ability. The wavelet energy (Ene_800nm_) was calculated as follows:


(5) $$E\,n\,e_{800\,n\,m}=\frac{1}{9}\,\sum_{i=1}^{3}\sum_{j=1}^{3}{\left|f\,\left(x,y\right)\right|}^{2}$$

where *f*(*x*,*y*) is the value of the LL image decomposed by wavelet transform of the 800 nm reflectance image.

The near-infrared (NIR) spectral characteristics of the crop are related to the canopy structure of the crop, and the reflectivity changes more obviously than that of visible bands. Therefore, the Ene_800 *nm*_ obtained from the 800 nm reflectance images contained abundant texture information reflecting the canopy structure. Some studies have also proved the potential of texture features including NIR bands in biological parameter estimation (Colorado et al., 2020; Lu et al., 2021). Multiplication is a common feature combination method in the estimation of crop growth parameters (Badgley et al., 2017; Zeng et al., 2019, 2021; Wang et al., 2020; Hao et al., 2021). To highlight the target leaf information and limit the influence of the soil background and panicles, the plot-level values of VI and Ene_800 *nm*_ were multiplied to obtain a new index, recorded as WT-VI. A total of 12 WT-VIs were calculated according to the corresponding VIs (Figure 3).

Wavelet texture features were used as supplementary information of spectral features to broaden the data dimension of UAV images to improve the accuracy of rice LAI estimation. The performance of the calculated WT-VIs in the estimation of rice LAI was evaluated and compared with the traditional VIs in this study.

### Model validation

The final model of rice LAI estimation was established using the method of k-fold cross-verification in this experiment. It divided all samples into k disjoint sets (*k* = 10 in this study). Each time, one of the subsets is used as the test set and the other k-1 sets as the training set. The k-1 sets are repeatedly applied to calibrate the coefficients (Coef_*i*_) and coefficients of determination (*R*^2^_*i*_) for the established algorithm. We repeat the aforementioned steps k times, and the average values of root mean square error (RMSE) and relative root mean square error (rRMSE) based on the test set will be obtained (Peng et al., 2019):


(6) C⁢o⁢e⁢f2=1k⁢∑i=1kC⁢o⁢e⁢fi2;R2=1k⁢∑i=1kRi2;R⁢M⁢S⁢E=1k⁢∑i=1kR⁢M⁢S⁢Ei,r⁢R⁢M⁢S⁢E=1k⁢∑i=1kr⁢R⁢M⁢S⁢Ei

The training process of the model is repeated k times, and each sample is used for one validation, allowing the model to be optimized.

## Result

### Relationships between vegetation index and leaf area index of rice

**FIGURE 4:**
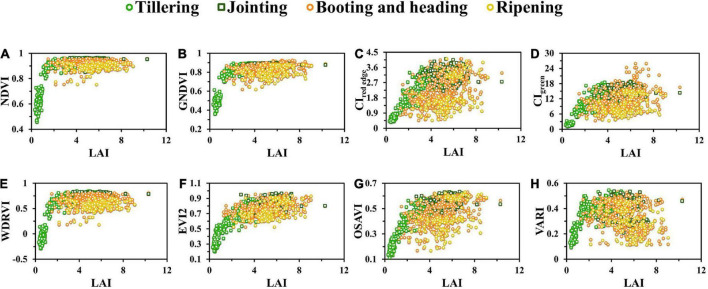
Variation of LAI plotted against vegetation indices: **(A)** NDVI, **(B)** GNDVI, **(C)** CI_*red  edge*_, **(D)** CI_*green*_, **(E)** WDRVI, **(F)** EVI2, **(G)** OSAVI, and **(H)** VARI. The scattered points were marked with four colors according to the growth stages of rice: tillering, jointing, booting and heading, and ripening.

To explore the relationships between rice LAI and VIs based on UAV images, we established the linear regression models of the VI and LAI and obtained R^2^, as shown in Table 4. The linear regression model was the most common model for crop parameter estimation (Jiang et al., 2019). Figure 4 presents the scatter plots of the VI and rice LAI, which reflected the variation of the VI with LAI throughout the growing season. The distribution of the VI in the two study areas was similar. The scattered plots were marked with four colors according to the growth stages of rice: tillering, jointing, booting and heading, and ripening. The results in Table 4 indicated that R^2^ values for all VIs throughout the entire growth period of rice were very low, not more than 0.4 except for OSAVI and EVI2, and VARI particularly was the lowest (R^2^ was less than 0.1). Among all tested VIs, EVI2 and OSAVI held the highest R^2^ values of 0.426 and 0.540 (Table 4), respectively. The scattered points between pre-heading and post-heading stages of the ratio indices (CI_*red  edge*_ and CI_*green*_) (Figures 4C,D) and VARI were severely separated, of which VARI was most separated (Figure 4H), while that of EVI2 was slightly weaker (Figure 4F).

**TABLE 4:** R^2^ of the linear regression of rice LAI with VIs.

VI	VARI	WDRVI	SR	CI_*green*_	NDVI	MTCI	CI_*red edge*_	RVI	NDRE	GNDVI	OSAVI	EVI2
R^2^	0.020	0.307	0.315	0.315	0.316	0.334	0.335	0.335	0.366	0.368	0.426	0.540

As shown in Figure 4, when LAI was higher than 3, the sensitivity of all VIs to LAI decreased and saturation occurred, with normalized indices (NDVI and GNDVI) (Figures 4A,B) being the most obvious, which reduced the monitoring accuracy of the established model.

### Estimation of rice leaf area index with texture features

**FIGURE 5:**
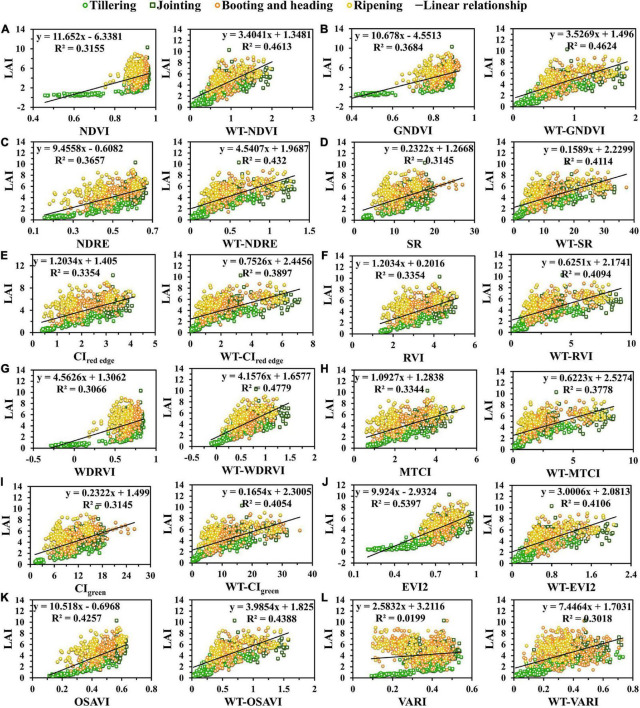
Two models of VIs and WT-VIs: **(A)** NDVI, **(B)** GNDVI, **(C)** NDRE, **(D)** SR, **(E)** CI_*red  edge*_, **(F)** RVI, **(G)** WDRVI, **(H)** MTCI, **(I)** CI_*green*_, **(J)** EVI2, **(K)** OSAVI, and **(L)** VARI. The scattered points were marked with four colors according to the growth stages of rice: tillering, jointing, booting and heading, and ripening.

In this study, we used WT-VIs to estimate rice LAI. VIs and WT-VIs were used to establish linear models with LAI, respectively. The fitting results of several VIs and WT-VIs are shown in Figure 5, and the performance of LAI estimation was compared.

Among all VIs, the scatter points in WT-VIs after heading were less separated than those in initial VIs. Compared with the VI, the WT-VI was less affected by the saturation effect. For the normalized indices (NDVI, GNDVI, and NDRE) in Figures 5A–C, the saturation effect of the original VIs began to appear when LAI increased to a certain extent, which seriously affected the effect of LAI estimation, while the saturation effect of the corresponding WT-VIs had been significantly improved (Figure 5A). Combined with the wavelet texture feature Ene_800 *nm*_, the scatter points of the WT-NDVI after the heading stage became less separated. In addition, in terms of improving the separation effect before and after the heading stage, the performance of the ratio indices (CI_*red  edge*_ and CI_*green*_) (Figures 5E,I) was not as good as that of the normalized indices (Figure 5A). The scatter points of VARI after the heading stage showed an opposite trend to that before the heading stage, while the severe separation before and after the heading stage was greatly attenuated in the WT-VARI (Figure 5L).

From the scatter plots in Figure 5G, the scatter points of the WT-WDRVI and LAI were the closest to a straight line among all WT-VIs. It was obvious that the scatter after the heading stage was more discrete in the WDRVI, while the scatter in WT-WDRVI was near a straight line with that before the heading stage. On the whole, the linear regression models established by the WT-VI and LAI performed better than VIs, which were less affected by the saturation effect and the separation before and after the heading stage.

### K-fold cross-validation

In Table 5, the performances of rice LAI inversion with WT-VIs and VIs were compared by using the R^2^ and RMSE after 10-fold cross-validation. By using 10-fold cross-validation, the R^2^ of Ene_800 *nm*_ is 0.4022 and the RMSE is 1.4214. Most WT-VIs that contained texture information improved the accuracy of estimating LAI using VIs alone.

**TABLE 5:** Comparison of the accuracy of VI and WT-VI models using 10-fold cross-validation.

	VI		WT-VI	
	
	R^2^	RMSE	R^2^	RMSE
NDVI	0.301	1.552	0.444	1.376
GNDVI	0.362	1.491	0.454	1.373
NDRE	0.356	1.497	0.429	1.402
SR	0.311	1.550	0.396	1.441
CIred edge	0.324	1.529	0.373	1.471
CIgreen	0.297	1.555	0.391	1.444
WDRVI	0.285	1.560	0.466	1.353
MTCI	0.328	1.531	0.367	1.481
EVI2	0.515	1.271	0.405	1.439
OSAVI	0.405	1.422	0.416	1.425
RVI	0.317	1.529	0.397	1.443
VARI	0.009	1.858	0.292	1.567

**FIGURE 6:**
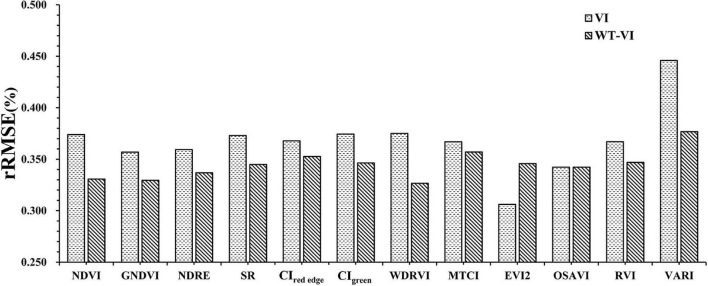
rRMSE of VI and WT-VI.

In addition, different types of VIs combined with texture features can improve the inversion effect of LAI. Compared with corresponding VI, R^2^ of the WT-VI increased by more than 0.15 (WT-NDVI, WT-WDRVI, WT-VARI) and RMSE decreased by more than 0.2 (WT-WDRVI, WT-VARI). The rRMSE of WT-VARI decreased by more than 6%, as shown in Figure 6. The estimation accuracy of the WT-WDRVI was the highest, with an R^2^ of 0.466 and an RMSE of 1.353. The inclusion of Ene_800 *nm*_ features did not significantly improve the accuracy of the models for OSAVI and EVI2, which were both the modified indices, and R^2^ and RMSE were not significantly improved.

**FIGURE 7:**
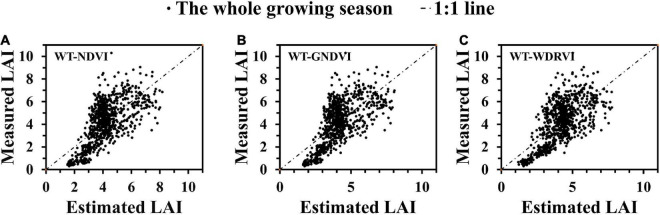
Relationships between measured LAI and estimated LAI. **(A)** WT-NDVI. **(B)** WT-GNDVI. **(C)** WT-WDRVI.

In general, the WT-VI-based model achieved higher accuracy than the model based on VIs for most indices. Based on the results of the model established by 10-fold cross-validation, we concluded that the models developed from the WT-WDRVI, WT-GNDVI, and WT-NDVI were the best models for estimating LAI of different rice varieties during the whole growing season. Therefore, we compared the relationships between the estimated LAI and the measured LAI, as shown in Figure 7, and the corresponding calculation formulas are as follows:


L⁢A⁢I=4.16×(W⁢T-W⁢D⁢R⁢V⁢I)+1.66,R⁢M⁢S⁢E=1.353,r⁢R⁢M⁢S⁢E=32.66%

L⁢A⁢I=3.53×(W⁢T-G⁢N⁢D⁢V⁢I)+1.50,R⁢M⁢S⁢E=1.373,r⁢R⁢M⁢S⁢E=32.94%

L⁢A⁢I=3.40×(W⁢T-N⁢D⁢V⁢I)+1.35,R⁢M⁢S⁢E=1.376,r⁢R⁢M⁢S⁢E=33.07%

## Discussion

The main objective of this study was to improve the estimation of rice LAI based on the UAV remote sensing images. We introduced the texture feature Ene_800 *nm*_ based on the wavelet transform of the NIR image to increase the LAI estimation accuracy and reduce the influence of the saturation effect and the serious separation before and after heading.

In this research, we selected 12 VIs (NDVI, GNDVI, NDRE, SR, CI_*red  edge*_, CI_*green*_, WDRVI, MTCI, EVI2, OSVAI, RVI, and VARI) to establish the model. These VIs had extensively been used in estimating LAI, aboveground biomass (Jiang et al., 2019), and yield of crops (Yuan et al., 2021). With the gradual growth of rice, all indices saturated rapidly before the heading stage, particularly for the normalized VIs such as NDVI and GNDVI. The scatter separation before and after the heading stage of VIs was severe for CI_*red  edge*_, VARI, and CI_*green*_. This may be due to the transformation of rice from vegetative growth to reproductive growth in the late growth stage, resulting in a slight decrease in the leaf area. After rice heading, the appearance of panicles changes the spectral reflectance of the canopy. At the same time, the leaves are withered and yellow, and the leaf area is also changing, so the accuracy of the model is affected. It can be seen from Figure 2 that the mean value of LAI was relatively stable from the jointing to the maturity stage, but the range of values was wide in the mature stage. At the same time, the vegetation indices were seriously saturated in high coverage conditions, which seriously affected the estimation effect of LAI in the late growth stage. Therefore, it was necessary to consider improving the LAI estimation effect at the late growth stage. Apparently, the VI only reflects spectral information and is deficient in its ability to describe the detailed texture features of the shape, size, and height of the crop canopy of images. The use of canopy reflectance and VI alone is not sufficient for LAI estimation. According to the characteristics of the vegetation spectral curve, the NIR images contain information on the vegetation canopy structure (Imran et al., 2020). Meanwhile, LAI calculated from the vegetation leaf area is an essential vegetation growth indicator for the canopy structure. Therefore, it was appropriate to use texture features based on NIR images to estimate LAI. To obtain the texture features of the remote sensing images of rice, the images were transformed from the spatial domain to the wavelet domain using the wavelet transform, and Ene_800 *nm*_ was calculated.

**FIGURE 8:**
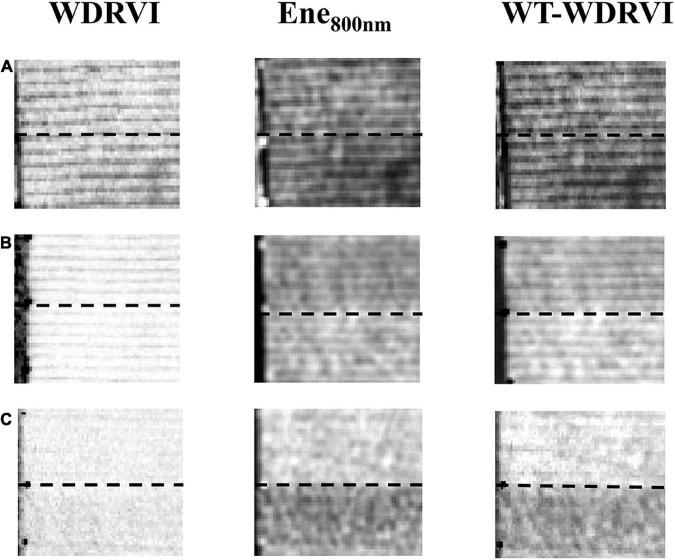
WDRVI, Ene_800 nm_, and WT-WDRVI images of two plots in different growth stages. **(A)** DAT 17, at the early tillering stage. **(B)** DAT 47, at the late tillering stage. **(C)** DAT 78, at the ripening stage. To better describe these features, two typical plots are selected to display. With the dotted line as the boundary, the upper field block is plot 1 and the lower field block is field plot 2.

**FIGURE 9:**
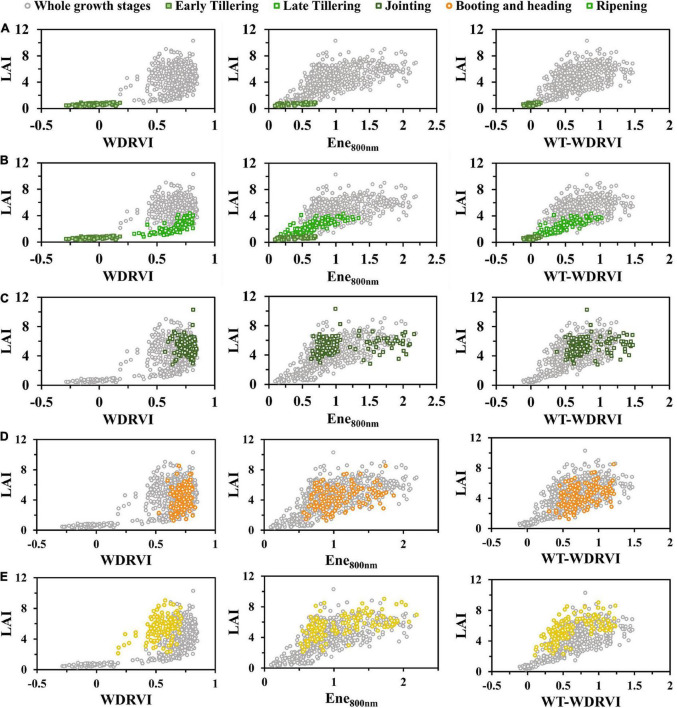
WDRVI vs LAI, Ene_800 nm_ vs LAI, and WT-WDRVI vs LAI of whole growth stages. **(A)** Early tillering. **(B)** Late tillering. **(C)** Jointing. **(D)** Booting and heading. **(E)** Ripening. To highlight the difference in texture features, the early tillering and late tillering stages are displayed separately.

We combined texture features with the VI and built new models to improve the rice LAI estimation. The WT-VI can highlight the leaf information at different growth stages of rice and weaken the interference of the soil background and panicles. The complex canopy structural changes that were not well described by VI could be well described by texture features. Almost all VIs combined with the texture features could improve the estimation of rice LAI with similar patterns, and the effect of the WDRVI was more obvious and intuitive. Therefore, taking WDRVI as an example, VI, Ene_800 *nm*_, and WT-WDRVI images in different growing stages are shown in Figure 8. The effects of the VI and WT-VI on LAI estimation were compared in detail in different growth stages in Figure 9.

In the tillering stage, it was observed from the scatter plot (Figure 9B) that the relationship between the VI and LAI was exponential and that between Ene_800 *nm*_ and LAI was linear. The scatter points of the WT-VI and LAI tended to approach a straight line. In Figure 9A, LAI was at a low level in the early tillering stage, and the values of the VI and Ene_800 *nm*_ were both small and less than 1. The values of the VI multiplied by Ene_800 *nm*_ were even smaller, and the corresponding scatter points would be more concentrated. The combination of the VI and texture features weakened the saturation effect of the VI in the late tillering stage. Texture features played a complementary role to the VI, which made the index of WT-VI very sensitive to LAI in the early stage of rice growth and had a strong linear relationship with LAI. Many studies have shown that the effect of the soil background is very significant at the initial stage of rice growth and decreases with the increase in vegetation coverage (Sun et al., 2017).

In the early tillering stage, the leaves of rice were smaller and the vegetation coverage was lower for irrigated paddy fields. Water and soil background significantly affected the plant canopy reflectance. Even when LAIs were similar, the differences of VIs were also large (Figure 9A). Ene_800 *nm*_ images blurred the edges of leaves and soil. As can be seen from the WT-VI image (Figure 8A), the bright lines were the leaves, and the dark lines were the soil background. The leaf information was highlighted clearly, and the effect of soil background was greatly diminished. By the late tillering stage, rice continued to grow and the leaves gradually became larger and more numerous. The difference between the two plots was small in the WDRVI image (Figure 8B) but obvious in the Ene_800 *nm*_ image (Figure 8B). Ene_800 *nm*_ was still sensitive to LAI, and the WT-VI greatly reduced the saturation effect.

In the jointing stage, it was observed from the scatter plot (Figure 9C) that LAI of rice increased rapidly, and the VI was obviously saturated. The values of LAI corresponding to similar VIs were quite different. In the late growth stage of rice, LAI was at a high level, and the WDRVI was already saturated, with values clustered to a small range. However, under the high vegetation coverage conditions, Ene_800 *nm*_ was not saturated, and the results of the multiplication of the VI and Ene_800 *nm*_ were mainly affected by the texture features and retained the sensitivity to LAI. As rice grew, the background of the soil was obscured by larger and more leaves. The differences in the canopy structure (leaf inclination, size, position distribution, etc.) were difficult to be accurately reflected by the VI, while the texture features could better describe it. When the canopy of rice had closed, the addition of texture features reduced the saturation effect of the VI.

After rice heading, the appearance of panicles, and the yellowing and falling off of the leaves at the bottom of the canopy made the canopy structure more complex, which could affect the light distribution within the rice canopy and interfere with the spectral features of the canopy. Therefore, the VI contained not only the information on leaves but also the information on rice panicles. In the scatter diagram (Figures 9D,E), the VI results were relatively discrete. The texture features were less affected by the panicles, and the scatter points of Ene_800 *nm*_ with LAI were not so discrete. After the heading stage, the performance of LAI estimation by the WT-VI obtained by multiplying the VI and Ene_800 *nm*_ was more influenced by Ene_800 *nm*_. In Figure 8C, plot 1 in the Ene_800 *nm*_ texture image was relatively brighter with a larger leaf-to-panicle ratio, while plot 2 was darker with more panicles, and the panicles were drooping and cluttered. In the image of the WT-VI, plot 1 became brighter, and the leaf information was more prominent when the panicles and leaves were mixed, so the panicles were suppressed. While plot 2 became darker, with the panicle being weakened. However, in the late stage of rice growth, the leaves of a small part of the fields withered and fell seriously. When the leaf-to-panicle ratio decreased, the inhibition of Ene_800 *nm*_ on panicles was not obvious, and there were some discrete points in Figure 9E, which led to the limited improvement.

In this study, the estimation accuracy of the WT-WDRVI was the highest in the whole growth period of rice (Table 5). The addition of Ene_800 *nm*_ can significantly improve the estimation effect of the normalized indices (NDVI, GNDVI, and NDRE) on LAI (Figure 6). The reason is that the normalized indices are susceptible to the influence of soil background and panicles, which can be weakened by the addition of Ene_800 *nm*_. The WT-VI was highly sensitive to LAI at the key growth stages of complex changes in the rice canopy structure, especially in the early tillering, late tillering, and post-heading stages. The improvement effect of texture features on the VI before the heading stage was better than that after the heading stage (Figure 9). The VI is the most widely used method to estimate LAI, and texture features are used to assist the VI to improve the accuracy of LAI estimation. The proposed method has an enhancement effect on most VIs. However, for the modified indices (EVI2), the improvement effect was not as good as the normalized indices given in Figure 5J. Neither R^2^ nor RMSE had improved significantly (Table 5). OSAVI, which is the soil-adjusted VI, can reduce the effect of soil reflectance at low LAI. EVI2 is a 2-band version of the EVI that does not include the blue band, which retains the sensitivity of the EVI to high LAI vegetation (Liu et al., 2012). EVI2 has been found to remain sensitive to a wider range of LAI and resist to changes in soil background reflectance and atmospheric conditions (Jiang et al., 2008). The modified indices are less affected by the soil background than the normalized indices (Figure 4), so using only the modified indices to estimate LAI can get satisfactory results. Precisely for this reason, the improvement effect of our method on the modified indices is limited.

In this study, we used the 800 nm band for experiments. In addition, we also found that texture features of other NIR bands of the Mini-MCA 12 multispectral camera, except 800 nm, could also achieve similar accuracy, among which 900 nm had the best effect. NIR bands are related to the canopy structure of the vegetation. Therefore, the texture features extracted from NIR band images contain rich information related to the canopy structure. In addition, considering the R^2^ and RMSE of VI, WT-VI, and Ene_800 *nm*_ in Table 5, it can be found from Figure 9 that the performance of the WT-VI estimation of LAI depended on the texture features extracted from the NIR band images. Thus, although we only used 800 nm in this study, similar results could be achieved for all NIR bands of MCA.

In general, the WT-VI performed better than the VI in LAI estimation, with higher R^2^ and lower RMSE and rRMSE (Figure 6), especially the normalized indices (NDVI, NDRE, and GNDVI). Wavelet texture features remained sensitive to the rice canopy structure, which varied greatly at different growth stages. The WT-VI reduced the effects of the soil background and panicles, which emerged at certain stages. The combination of texture information and spectral information weakened the saturation effect and the serious separation before and after the heading stage. The method proposed in this article can adapt to the multi-temporal differences in the rice canopy structure and avoid the time-consuming and laborious work of establishing models in each growth period. Compared with the physical model, the approach needs fewer parameters. The method we proposed in this article can be satisfactorily applied in precision agriculture, as well as for fields containing many breeding varieties. The model developed in this study was tested only on data from the two experiments together. However, this study provides a good example of applying remote sensing technology to accurately predict rice LAI, which has great potential in precision agriculture. Our future work is to apply this approach to different experiments to explore the transferability of the model and its robustness to factors such as species and image resolution.

## Conclusion

In this study, we developed a method for LAI estimation by combining spectral features and wavelet texture features, given the differences in the rice canopy structure in different growth stages. For most VIs, compared with using the VI alone, the combination of the VI and Ene_800 *nm*_ can estimate rice LAI more accurately. The RMSE and rRMSE of linear fitting of the WT-NDVI decreased to 1.270 and 32.6%, respectively. Since texture features have high sensitivity to the canopy structure of rice in different growth stages, combining spectral features with texture features can improve the accuracy of rice LAI estimation. The combination of texture features and spectral features weakens the effects of the soil background and panicles and reduces the saturation effect when the rice canopy closes. Thus, the method we proposed can be well applied in precision agriculture and field management. In our future study, we will explore the transferability of the model and identify additional factors that may affect the LAI estimation. Other texture features can be considered to improve the LAI estimation of rice for the unique and complex canopy structure changes of rice.

## Data availability statement

The original contributions presented in the study are included in the article/supplementary material, further inquiries can be directed to the corresponding author/s.

## Author contributions

YG and CZ provided the main research ideas and gave guidance at critical moments. CZ conducted image processing and data analysis and completed the main experiments. KY provided important experimental and writing guidance. SF and RZ supplied professional equipment and experimental sites. XW and RZ provided advice from an agronomic point of view. CZ and YG completed this manuscript together. All authors agreed with the manuscript and contributed significantly to this manuscript.
